# Exogenous glomalin boosts kumquat seedling growth by enhancing soil structure and biochemical activity

**DOI:** 10.3389/fpls.2025.1668905

**Published:** 2025-10-13

**Authors:** Yong Hao, Wei Lu, Chun-Yan Liu, Jiadong He

**Affiliations:** ^1^ College of Urban Construction, Yangtze University, Jingzhou, Hubei, China; ^2^ College of Horticulture and Gardening, Yangtze University, Jingzhou, Hubei, China; ^3^ Earth and Life Institute, Université Catholique de Louvain-UCLouvain, Louvain-la-Neuve, Belgium

**Keywords:** aggregates, enzyme activity, glomalin-related soil protein (GRSP), organic carbon, root architecture, soil aggregation

## Abstract

**Introduction:**

Glomalin-related soil protein (GRSP), a nitrogen-linked glycoprotein secreted by arbuscular mycorrhizal fungi, is recognized for its role in enhancing soil physical and chemical properties and improving ecosystem stability. However, the dose-dependent effects of exogenous easily extractable GRSP (EE-GRSP) on perennial fruit crops such as citrus remain largely unexplored.

**Methods:**

The effects of varying strengths (1/4, 1/2, 3/4, and full strength (0.027 mg·mL^–1^)) of exogenous EE-GRSP on kumquat (*Fortunella japonica*) seedling growth, root architecture, and rhizosphere soil properties (enzyme activities, aggregate stability, organic carbon content, and GRSP content) were investigated.

**Results:**

EE-GRSP significantly promoted kumquat growth and root development, with the 3/4 strength (0.020 mg·mL^–1^) exhibiting pronounced positive effects on plant biomass and optimized root architecture. Concurrently, this optimal dosage markedly enhanced soil aggregate stability (mean weight diameter +92.9%) and stimulated key rhizosphere enzyme activities (up to 64%), correlating with increased soil organic carbon and de novo GRSP content. These improvements followed a parabolic dose-response, with excessive full-strength EE-GRSP diminishing benefits compared to the 3/4 dose.

**Discussion:**

These findings unequivocally establish that exogenous EE-GRSP can effectively promote citrus growth primarily by fostering robust synergistic soil-plant feedbacks through concomitant improvements in both soil physical structure (e.g., aggregate stability) and biochemical processes (e.g., enzyme activity and carbon sequestration). This study advances our mechanistic understanding of glomalin-mediated soil-plant interactions and highlights EE-GRSP as a promising and valuable soil amendment for sustainable citrus cultivation, urging further validation under diverse field conditions.

## 1 Introduction

The intricate interactions within the soil-plant system are fundamental to agricultural productivity and ecosystem stability (He et al., 2020). Among the myriad of soil microorganisms, arbuscular mycorrhizal (AM) fungi establish a ubiquitous symbiotic relationship with approximately 72% of terrestrial plants, playing a pivotal role in enhancing plant fitness and soil health (Brundrett and Tedersoo, 2018). These beneficial fungi are widely recognized for their capacity to improve root system architecture, augment water and nutrient uptake, and bolster plant resilience against both biotic and abiotic stresses (Cheng et al., 2024). Beyond direct plant benefits, AM fungi significantly influence the rhizosphere by secreting various organic compounds, thereby contributing to crucial ecosystem services such as mitigating soil erosion, chelating heavy metals, enhancing soil carbon sequestration, and stabilizing soil macroaggregates (Fall et al., 2022).

A key determinant of AM fungal-mediated benefits is glomalin-related soil protein (GRSP), a nitrogen-linked glycoprotein secreted by AM fungal spores and mycelium into the soil. GRSP is a complex macromolecule, primarily composed of proteins and carbohydrates, and contains diverse elements, including carbon, nitrogen, potassium, iron, and calcium, along with functional groups such as aromatic and carboxyl groups (Li et al., 2023; Nthebere et al., 2025). Based on extraction difficulty, GRSP is typically fractionated into easily extractable GRSP (EE-GRSP) and difficultly extractable GRSP (DE-GRSP), with their combined concentration referred to as total extracted GRSP (T-GRSP) (Wu et al., 2015). EE-GRSP represents the newly synthesized and relatively labile fraction, while DE-GRSP is a more recalcitrant form, derived from the turnover of EE-GRSP, persisting longer in the soil as a stable, inactive protein (Zou et al., 2016). Accumulating evidence highlights a strong positive correlation between AM fungal-induced plant growth improvements and GRSP secretion, primarily through enhanced soil aggregation, improved soil carbon storage, and increased plant stress tolerance (Hou et al., 2022). GRSPs are often considered analogues of humic substances due to their significant role in soil stabilization (Gu et al., 2024).

In recent years, the application of exogenous EE-GRSP as a potential biofertilizer has garnered considerable research attention. For instance, Meng et al. (2021) observed that foliar application of exogenous EE-GRSP to late-ripening sweet oranges significantly promoted plant growth and fruit quality, likely through enhanced root mycorrhizal colonization and increased soil hyphal length. Similarly, Wu et al. (2023a) demonstrated that EE-GRSP application improved tea plant quality, including carbohydrate, polyphenol, amino acid, catechin, and flavonoid content, attributing these effects to improved water absorption and transport mediated by aquaporins (AQPs). Furthermore, the growth-promoting effects of EE-GRSP are also implicated in improving soil structure, facilitating nutrient absorption, and optimizing root system architecture. In trifoliate orange seedlings, exogenous EE-GRSP significantly promoted the formation and stabilization of soil water-stable aggregates (WSAs), increased soil organic carbon (SOC) content, and enhanced soil phosphatase activity, all contributing to improved plant growth and biomass (Liu et al., 2021).

Despite these promising observations, a critical gap persists in fully elucidating the precise mechanisms by which exogenous EE-GRSP directly promotes plant growth, particularly concerning its specific roles in improving soil physical structure and modulating rhizosphere enzyme activities. The stability of WSAs is a crucial indicator of soil structural integrity, directly influencing soil aeration, water permeability, root development, SOC stabilization, and resistance to erosion (Nthebere et al., 2024; Yan et al., 2024). WSA formation is a complex process influenced by factors including soil organic matter (SOM), plant roots, microbial communities, and their secreted metabolites (Chen et al., 2022; Gałązka et al., 2020). Previous studies suggest that AM fungi enhance WSA formation through both physical entanglement by extensive hyphal networks (Wang et al., 2022) and chemical stabilization via GRSP secretion, which modifies aggregate size distribution and improves soil-water relationships (Wu et al., 2023b; Zou et al., 2014). Strong positive correlations between GRSP concentration and WSA percentages have been established (Fokom et al., 2012; Nthebere et al., 2025). For citrus, foliar spraying of exogenous EE-GRSP has been shown to improve WSA distribution (0.25–2 mm grain size), mycelium length, and GRSP concentrations (Liu et al., 2022b), and purified EE-GRSP positively impacted soil properties, promoting GRSP content, nutrient availability, and WSA stabilization in citrus orchards (Zheng et al., 2024). Concurrently, the activity of soil enzymes, such as peroxidase (POD), phosphatase, and polyphenol oxidase (PPO), is indispensable for the biogeochemical cycling of carbon, nitrogen, and phosphorus, all vital for plant growth (Nthebere et al., 2024). Given GRSP’s organic carbon and nitrogen composition, exogenous EE-GRSP is hypothesized to directly influence soil enzyme activities. However, whether the observed plant growth-promoting effects of GRSP are a direct consequence of improved soil structure and enhanced enzyme activities remains an unexplored mechanistic link.

To address this critical knowledge gap, this study sought to identify an optimal application concentration of exogenous EE-GRSP for maximizing kumquat seedling growth and to elucidate the underlying soil-plant synergistic mechanisms. It is hypothesized that exogenous EE-GRSP enhances kumquat growth by synergistically improving both soil physical and chemical properties. To test this hypothesis, kumquat seedlings were exposed to varying EE-GRSP concentrations under controlled conditions. Plant responses were comprehensively evaluated through assessments of root system architecture, plant biomass, rhizosphere soil enzyme activities, soil aggregate distribution, and the content of SOC and GRSP fractions. By defining this dose-response relationship, this findings provide crucial mechanistic insights into the functional significance of exogenous GRSP in soil-plant interactions, ultimately contributing to the development of sustainable soil amendments for improved citrus performance in agricultural systems.

## 2 Materials and methods

### 2.1 Preparation of exogenous EE-GRSP solution

Soil for extracting EE-GRSP was collected from the rhizosphere of a 13-year-old citrus orchard located at Yangtze University, Jingzhou, Hubei Province, China (30.36° N, 112.15° E), at a depth of 0–20 cm. The basic physico-chemical properties of this soil were as follows: pH 5.8, available nitrogen 101.8 mg·kg^-1^, available phosphorus 22.38 mg·kg^-1^, and available potassium 94.48 mg·kg^-1^ (Liu et al., 2020a). The soil was well mixed, air-dried, and sieved through a 4mm mesh to remove roots, litter, and stones. EE-GRSP was extracted according to the method described by Koide and Peoples (2013). Soil samples were extracted with 20 mmol·L^−1^ citrate buffer (pH 7.0) at 0.1 MPa and 121°C for 30min. The supernatant was collected as the stock solution, referred to as full-strength EE-GRSP, with a concentration of 0.027 mg·mL^−1^ protein, as determined based on our previous study on citrus rhizosphere soil (Wu et al., 2023a; Wu et al., 2023b). The stock solutions were stored at 4°C for less than one month.

### 2.2 Experimental design

A completely randomized design was employed, with varying strengths of EE-GRSP as the experimental factors. The design included five treatments: citrate buffer solution (20 mmol·L^−1^, pH 7.0), quarter-strength EE-GRSP solution (1/4 EE-GRSP), half-strength EE-GRSP solution (1/2 EE-GRSP), three quarters-strength EE-GRSP solution (3/4 EE-GRSP), and full-strength EE-GRSP solution (full EE-GRSP). The EE-GRSP solutions at 1/4, 1/2, and 3/4 strength were prepared by diluting the full-strength EE-GRSP solution with a certain proportion of 20 mmol·L^−1^ (pH 7.0). Each treatment was replicated five times, for a total of 25 pots.

### 2.3 Plant culture

Twenty-day-old kumquat (*Fortunella japonica* (Thunb.) Swingle) seedlings, free of mycorrhizal infection and of similar size with two leaves, were transplanted into 2 L plastic pots containing 1.5kg of autoclaved (0.11 MPa, 121°C, 1h) yellow loam soil from same citrus orchard. All seedlings were placed in a greenhouse at Yangtze University (Jingzhou) under controlled conditions: a photon flux density of 900 µmol·m^–2^·s^–1^, a diurnal temperature range of 28/20°C (day/night), and a relative humidity of 80%. During the first four weeks, the plants were maintained by watering 80 mL of distilled water per pot every two days. Starting from the fifth week, 80 mL of the respective EE-GRSP solutions was applied weekly for four weeks (weeks 5-8), totaling four applications. Distilled water was added as needed to maintain soil moisture during the EE-GRSP treatment. The plants were harvested after twelve weeks.

### 2.4 Variable determinations

The fresh weights of shoots and roots were measured the same day after harvested. The entire root systems of the kumquat seedlings were scanned using an Epson Perfection V700 Photo Dual Lens System (J221A, Seiko Epson Corporation, Jakarta Selatan, Indonesia), and root morphology was analyzed using Win RHIZO. The tap root length and the number of lateral roots were measured manually. The rhizosphere soil was collected, air-dried and sieved through a 4mm mesh screen for subsequent determination of soil indicators.

The activities of soil PPO and POD were determined according to the method of Almeida et al. (2019). The assay is based on the incubation of soil with pyrogallic acid as a substrate. For POD activity, hydrogen peroxide was additionally supplied.

Soil phosphatase activity was determined using sodium phenyl phosphate method described by Zhao and Jiang (1986). Briefly, soil samples were incubated with disodium phenyl phosphate as a substrate. The activities of acid, neutral, and alkaline phosphatase were distinguished by using specific buffer solutions at pH 5.0, 7.0, and 10.0, respectively. The amount of phenol released was determined colorimetrically at 570 nm.

The distributions of WSAs in the size fractions of 2–4 mm, 1–2 mm, 0.5–1 mm, and 0.25-0.5mm sizes were determined using the wet-sieving method (Rieke et al., 2022) with a soil aggregate analyzer (DM200-IV, Shanghai, China). The percentage of aggregates in each particle size was calculated, and the soil WSAs stability was expressed as the mean weight diameter (MWD), calculated according to the formula (Rieke et al., 2022):


$$MWD=\sum_{i=1}^{n}\text{WiXi}$$

Where, *n* is number of size fractions, *W_i_* is the proportion of the *i* size fraction in the total sample mass, *X_i_* is the mean diameter of the *i* sieve opening (mm). The MWD serves as an indicator of soil structural stability, with higher values indicating better soil aggregate stability.

Rhizosphere soil was collected to determine GRSP and soil organic carbon (SOC) contents. EE-GRSP and DE-GRSP were extracted according to the method of Koide and Peoples (2013). In brief, EE-GRSP was extracted with a mild citrate buffer (20 mM, pH 7.0) at 121°C for 30min. The remaining soil residue was then subjected to a harsher extraction with a higher pH citrate buffer (50 mM, pH 8.0) at 121°C for 60min to obtain DE-GRSP. Protein concentrations in both fractions were determined using the Bradford assay with bovine serum albumin as the standard Bradford (1976). Total GRSP (T-GRSP) was the sum of EE-GRSP and DE-GRSP. SOC was determined by the dichromate oxidation spectrophotometric method (Rowell, 2014).

### 2.5 Statistical analysis

Data processing and graph creation were performed using Microsoft Excel 2021 (Microsoft Corporation, Redmond, WA, USA) and SigmaPlot version 10.0 (Systat Software Inc., San Jose, CA, USA). Statistical analyses were conducted using SAS version 9.1.3 (SAS Institute Inc., Cary, NC, USA). One-way analysis of variance (ANOVA) was employed to determine the significance of differences among treatments, followed by Duncan’s multiple range test for multiple comparisons at a significance level of *P*<0.05. Pearson correlation coefficients were calculated using SAS software to assess relationships between variables, with graphs generated in SigmaPlot 10.0. No generative artificial intelligence (AI) or AI-assisted tools were used to create or alter images, figures, or artwork in this study.

## 3 Results

### 3.1 Biomass

**Figure 1:**
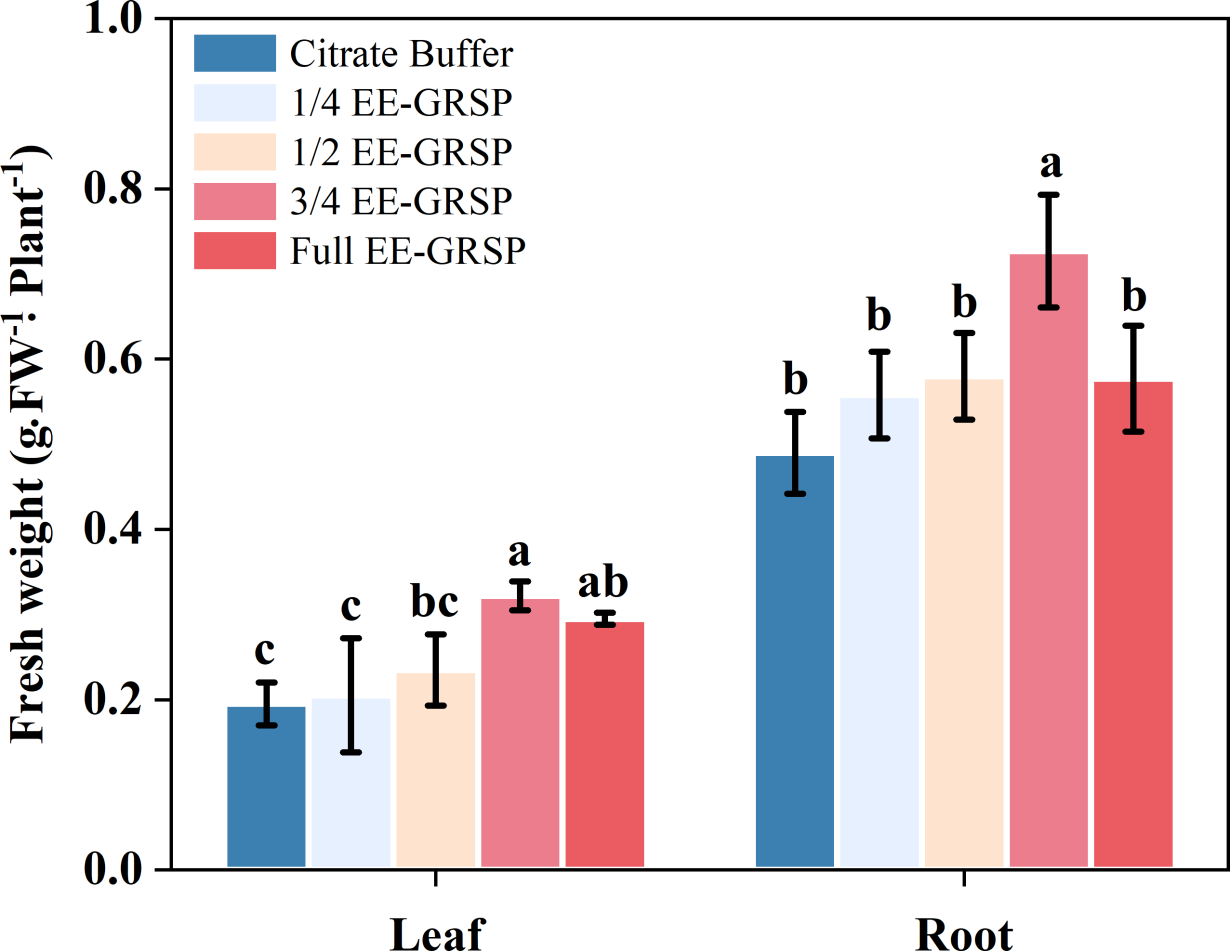
Effect of four different concentrations (1/4, 1/2, 3/4, and full) of exogenous easily extractable glomalin-related soil protein (EE-GRSP) on leaf and root fresh weight of kumquat seedlings. Data (mean ± SD, *n*=5) followed by different letters above the bars indicate significant differences (*P<* 0.05). Control, citrate buffer solution; 1/4, quarter-strength EE-GRSP; 1/2, half-strength EE-GRSP; 3/4, three-quarters-strength EE-GRSP; Full, full-strength EE-GRSP.

Application of 1/4 and 1/2 strength EE-GRSP treatments did not significantly affect leaf fresh weight of kumquat plants compared to the citrate buffer control. However, higher EE-GRSP concentrations (3/4 and full strength) significantly increased leaf fresh weight by 18.29% and 11.43%, respectively (**Figure 1**). No statistically significant difference was observed in leaf biomass between the 3/4 and full-strength EE-GRSP treatments.

All exogenous EE-GRSP concentrations enhanced root fresh weight, with increases of 13.88%, 18.36%, 48.37%, and 17.75% for 1/4, 1/2, 3/4, and full-strength treatments, respectively, relative to controls (**Figure 1**). The 3/4 EE-GRSP treatment demonstrated the most pronounced effect, significantly exceeding both lower concentrations (1/4 and 1/2) and the full-strength treatment. While the 1/2 EE-GRSP treatment statistically outperformed the 1/4 dilution, it showed no significant difference from the full-strength EE-GRSP treatment. Furthermore, the full-strength and 1/4 EE-GRSP treatments exhibited similar effects on root biomass, with no statistical distinction between them.

### 3.2 Root system architecture

**Figure 2:**
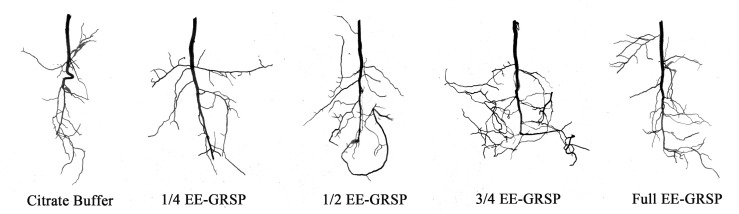
Root system architecture of kumquat seedlings under citrate buffer treatment and four different concentrations (1/4, 1/2, 3/4, and full) of exogenous extractable glomalin-related soil protein (EE-GRSP). Control: citrate buffer solution; 1/4: quarter-strength EE-GRSP; 1/2: half-strength EE-GRSP; 3/4: three-quarters-strength EE-GRSP; Full: full-strength EE-GRSP.

Exogenous EE-GRSP application exhibited differential effects on root system architecture parameters in kumquat plants (**Figure 2**). Compared to the citrate buffer control, the 1/4 EE-GRSP treatment significantly increased taproot length by 40.31%, but did not significantly affect other architectural parameters (**Table 1**). In contrast, higher EE-GRSP concentrations (1/2, 3/4, and full strength) induced substantial improvements across multiple parameters: total root length increased by 41.22%, 81.07%, and 36.30%, projected area by 30.47%, 54.97%, and 19.86%, and root volume by 40.87%, 66.81%, and 33.54%, respectively. These treatments also enhanced lateral root development, with first-order lateral roots increasing by 26.09%, 33.33%, and 26.09%, and second-order lateral roots increasing by 167.86%, 238.82%, and 42.01%, respectively (**Table 1**).

**Table 1:** Effect of our different concentrations (1/4, 1/2, 3/4, and full) of exogenous glomalin-related soil protein (EE-GRSP) on root system architecture of kumquat seedlings.

Treatments	Total length (cm)	Projected area (cm^2^)	Surface area (cm^2^)	Average diameter (mm)	Volume (cm^3^)	Taproot length (cm)	Lateral root numbers (#/Plant)	
							1^st^-order	2^nd^-order
Citrate Buffer	80.17±3.84c	7.35±0.52d	10.36±0.09a	0.69±0.02b	62.52±4.17c	9.03±0.06d	15.33±1.53b	10.33±1.53d
1/4 EE-GESP	87.40±4.45c	8.00±0.70cd	11.16±1.42a	0.77±0.03ab	66.78±10.93c	12.67±1.15c	16.00±2.00b	10.67±0.58d
1/2 EE-GESP	113.22±9.72b	9.59±0.46b	12.44±1.43a	0.79±0.08ab	88.07±5.84b	14.30±1.71b	19.33±1.53a	27.67±4.62b
3/4 EE-GESP	145.16±5.16a	11.39±0.82a	12.75±2.22a	0.82±0.09a	104.29±10.75a	15.00±2.00a	20.33±1.53a	35.00±2.65a
1 EE-GESP	109.27±4.27b	8.8±0.64bc	11.87±0.22a	0.74±0.04ab	83.49±19.14b	13.83±1.26bc	19.33±1.53a	14.67±1.53c

Data (means ± SE, *n*=5) followed by different letters (a, b, c, d) among treatments indicate significant differences at *P*<0.05. Control, citrate buffer solution; 1/4, quarter-strength EE-GRSP; 1/2, half-strength EE-GRSP; 3/4, three-quarters-strength EE-GRSP; Full, full-strength EE-GRSP.

All EE-GRSP concentrations significantly increased tap root length compared to the control, with increases of 9.02%, 41.22%, 81.07%, and 36.30% for 1/4, 1/2, 3/4, and full-strength treatments, respectively. However, root surface area remained statistically unchanged across all the treatments. Notably, only the 3/4 EE-GRSP treatment significantly enhanced average root diameter, increasing it by 18.84% (**Table 1**).

### 3.3 Rhizosphere enzyme activities

**Figure 3:**
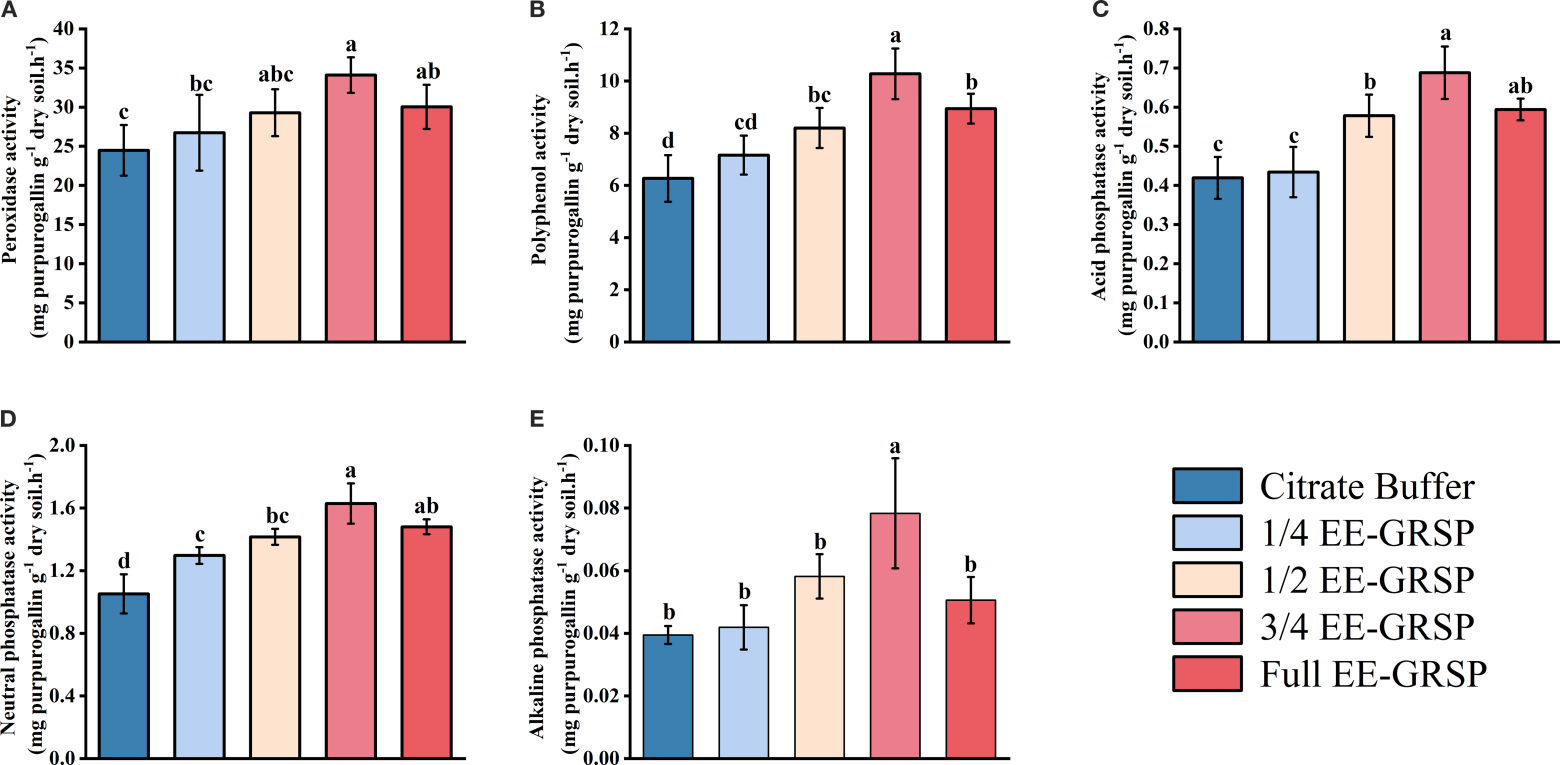
Effect of four different concentrations (1/4, 1/2, 3/4, and full) of exogenous easily extractable glomalin-related soil protein (EE-GRSP) on rhizosphere enzyme activities of kumquat seedlings. **(A)** Peroxidase (POD) activity, **(B)** Polyphenol (PPO) activity, **(C)** Acid phosphatase activity, **(D)** Neutral phosphatase activity, **(E)** Alkaline phosphatase activity. Data (mean ± SD, *n*=5) followed by different letters above the bars indicate significant differences (*P<* 0.05). Control, citrate buffer solution; 1/4, quarter-strength EE-GRSP; 1/2, half-strength EE-GRSP; 3/4, three-quarters-strength EE-GRSP; Full, full-strength EE-GRSP.

Exogenous EE-GRSP application significantly influenced rhizosphere soil enzyme activities, with the exception of alkaline phosphatase at the lowest concentration. Enzyme activities exhibited a consistent pattern of initial increase followed by decrease as EE-GRSP concentration increased, with peak values observed under the 3/4 EE-GRSP treatment (**Figure 3**). Compared to the citrate buffer treatment, EE-GRSP application significantly enhanced enzyme activities across multiple treatments. The PPO activity increased by 14.19%, 30.78%, 63.96%, and 42.58% in the 1/4, 1/2, 3/4, and full-strength EE-GRSP treatments, respectively (**Figure 3A**). The POD activity increased by 9.19%, 19.65%, 39.26%, and 22.71%, respectively (**Figure 3B**). Acid phosphatase activity increased by 3.58%, 37.95%, 46.15%, and 39.38%, respectively (**Figure 3C**), while neutral phosphatase activity increased by 25.00%, 35.24%, 55.24%, and 40.95%, respectively (**Figure 3D**). Alkaline phosphatase activity increased by 48.72%, 100.00%, and 30.77% in the 1/2, 3/4, and full-strength treatments, respectively, with no significant effect observed in the 1/4 EE-GRSP treatment (**Figure 3E**).

### 3.4 Distribution and stability of WSAs

Compared to the citrate buffer control, all four concentrations of EE-GRSP (1/4, 1/2, 3/4, and full strength) significantly increased the percentages of WSAs across multiple size fractions (2.00-4.00mm, 1.00-2.00mm, 0.50-1.00mm, and 0.25-0.50mm) and mean weight diameter (MWD) in the rhizosphere soil of kumquat seedlings (**Table 2**). The 3/4 EE-GRSP treatment consistently yielded the highest values for all measured parameters.

**Table 2:** Effect of four different concentrations (1/4, 1/2, 3/4, and full) of exogenous glomalin-related soil protein (EE-GRSP) on distribution of water stable aggregate (WSA) in the size ranges of 1–2 mm, 0.5–1 mm, and 0.25-0.5mm, and mean weight diameter (MWD) in the rhizosphere of kumquat seedlings.

Treatments	Percentage of WSAs (%)				MWD (mm)
	2–4 mm	1–2 mm	0.5–1 mm	0.25-0.5 mm	
Citrate Buffer	3.51±0.14d	9.57±0.47d	15.05±1.88d	16.24±1.81d	0.42±0.02d
1/4 EE-GESP	4.59±0.56c	12.51±1.51c	19.22±3.16c	20.63±1.71c	0.55±0.04c
1/2 EE-GESP	5.35±0.69b	14.61±0.50b	23.21±2.01b	23.63±0.82b	0.64±0.02b
3/4 EE-GESP	7.34±0.59a	17.98±1.69a	30.01±1.21a	25.09±2.86a	0.81±0.03a
1 EE-GESP	4.64±0.27c	14.13±1.21bc	25.28±1.98b	21.08±1.03bc	0.62±0.01b

Data (means ± SE, *n*=4) followed by different letters (a, b, c, d) among treatments indicate significant differences at *P*<0.05. Control, citrate buffer solution; 1/4, quarter-strength EE-GRSP; 1/2, half-strength EE-GRSP; 3/4, three-quarters-strength EE-GRSP; Full, full-strength EE-GRSP.

Specifically, the 1/4, 1/2, 3/4, and full-strength EE-GRSP treatments increased the percentage of WSAs in the 2.00-4.00mm fraction by 30.77%, 52.42%, 109.12%, and 32.19%, respectively. Similar patterns of enhancement were observed in other aggregate size fractions: 1.00-2.00mm WSAs increased by 30.72%, 52.66%, 87.88%, and 47.65%; 0.50-1.00mm WSAs increased by 27.71%, 54.22%, 99.40%, and 67.97%; and 0.25-0.50mm WSAs increased by 27.03%, 45.50%, 54.50%, and 29.80%, respectively. The MWD values followed a comparable trend, increasing by 30.95%, 52.38%, 92.86%, and 47.62%, respectively (**Table 2**).

**Figure 4:**
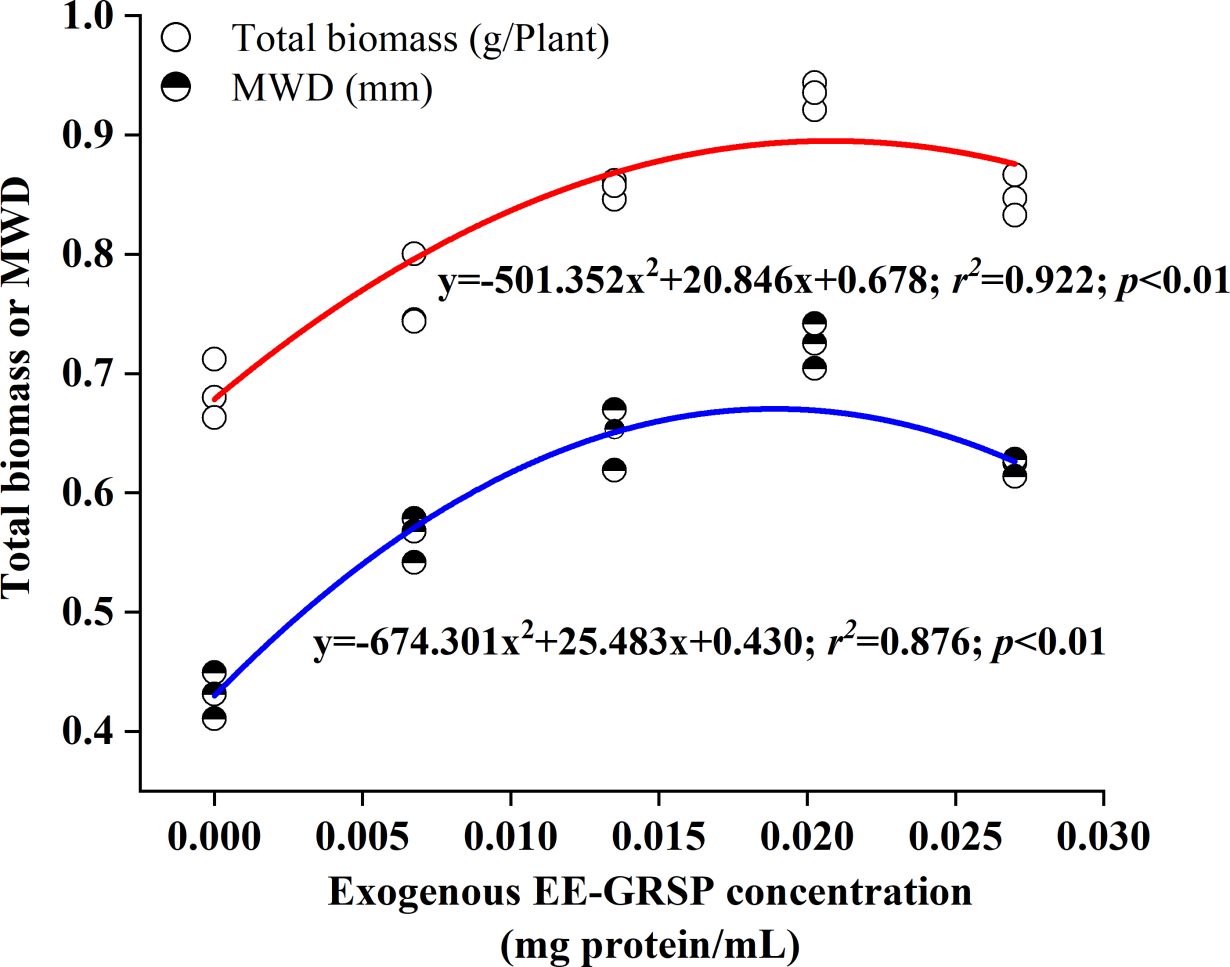
Quadratic regression between exogenous easily extractable glomalin-related soil protein (EE-GRSP) concentrations and total biomass (half-solid dots) or mean weight diameter (MWD) (open dots) in the rhizosphere of 3-month-old kumquat seedlings (*n*=25). Control, citrate buffer solution; 1/4, quarter-strength EE-GRSP; 1/2, half-strength EE-GRSP; 3/4, three-quarters-strength EE-GRSP; Full, full-strength EE-GRSP.

Quadratic regression analysis revealed a curvilinear relationship between MWD and GRSP concentration, characterized by two distinct phases (**Figure 4**). The first phase demonstrated a progressive increase in MWD from 0 to 0.020 mg·mL^–1^ protein citrate buffer (corresponding to the 3/4 EE-GRSP treatment), while the second phase exhibited a gradual decline in MWD from 0.020 to 0.027 mg·mL^–1^ protein citrate buffer (corresponding to the full EE-GRSP treatment).

### 3.5 GRSP and SOC content

The four concentrations of exogenous EE-GRSP significantly influenced the three fractions of GRSP and SOC concentration in the rhizosphere soil of kumquat seedlings. These parameters exhibited a consistent pattern of gradual increase with increasing exogenous GRSP concentration.

**Figure 5:**
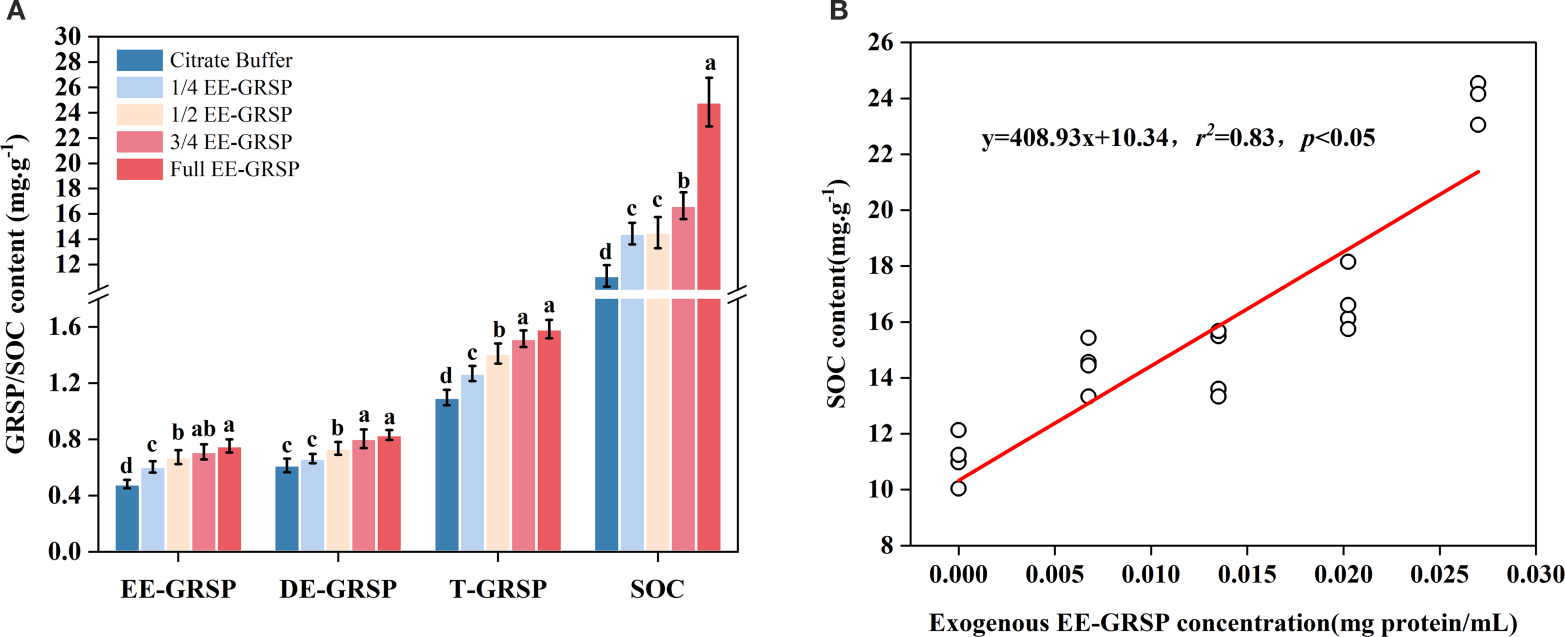
Effect of four different concentrations (1/4, 1/2, 3/4, and full) of exogenous easily extractable glomalin-related soil protein (EE-GRSP) on **(A)** soil organic carbon (SOC) concentration in kumquat seedlings and **(B)** linear regression between SOC and GRSP concentration in the rhizosphere soil of kumquat seedlings (*n* =25). Data (mean ± SD, *n*=5) followed by different letters above the bars indicate significant differences (*P<* 0.05). Control, citrate buffer solution; 1/4, quarter-strength EE-GRSP; 1/2, half-strength EE-GRSP; 3/4, three-quarters-strength EE-GRSP; Full, full-strength EE-GRSP.

Compared to the citrate buffer control, the 1/4, 1/2, 3/4, and full-strength EE-GRSP treatments increased EE-GRSP content by 27.08%, 39.58%, 47.91%, and 56.25%, respectively. Similarly, T-GRSP increased by 5.83%, 17.50%, 26.67%, and 31.67%, respectively. SOC content showed particularly dramatic increases of 30.09%, 30.90%, 50.09%, and 123.69%, respectively. In contrast, DE-GRSP exhibited significant increases only under the 1/2, 3/4, and full-strength EE-GRSP treatments, with increases of 12.06%, 29.03%, and 33.87%, respectively, while the 1/4 EE-GRSP treatment had no significant effect on DE-GRSP content (**Figure 5A**). Correlation analysis revealed a strong positive linear relationship between SOC content and exogenous EE-GRSP concentration (**Figure 5B**).

### 3.6 Pearson correlations

Correlation analysis revealed a significant associations between various parameters in the rhizosphere-plant system. The EE-GRSP content showed significant positive correlations with soil neutral phosphatase activity (*P*<0.05). The DE-GRSP and T-GRSP were strongly correlated with leaf fresh weight, polyphenol content, and both acid and neutral phosphatase activities (*P*<0.05).

**Figure 6:**
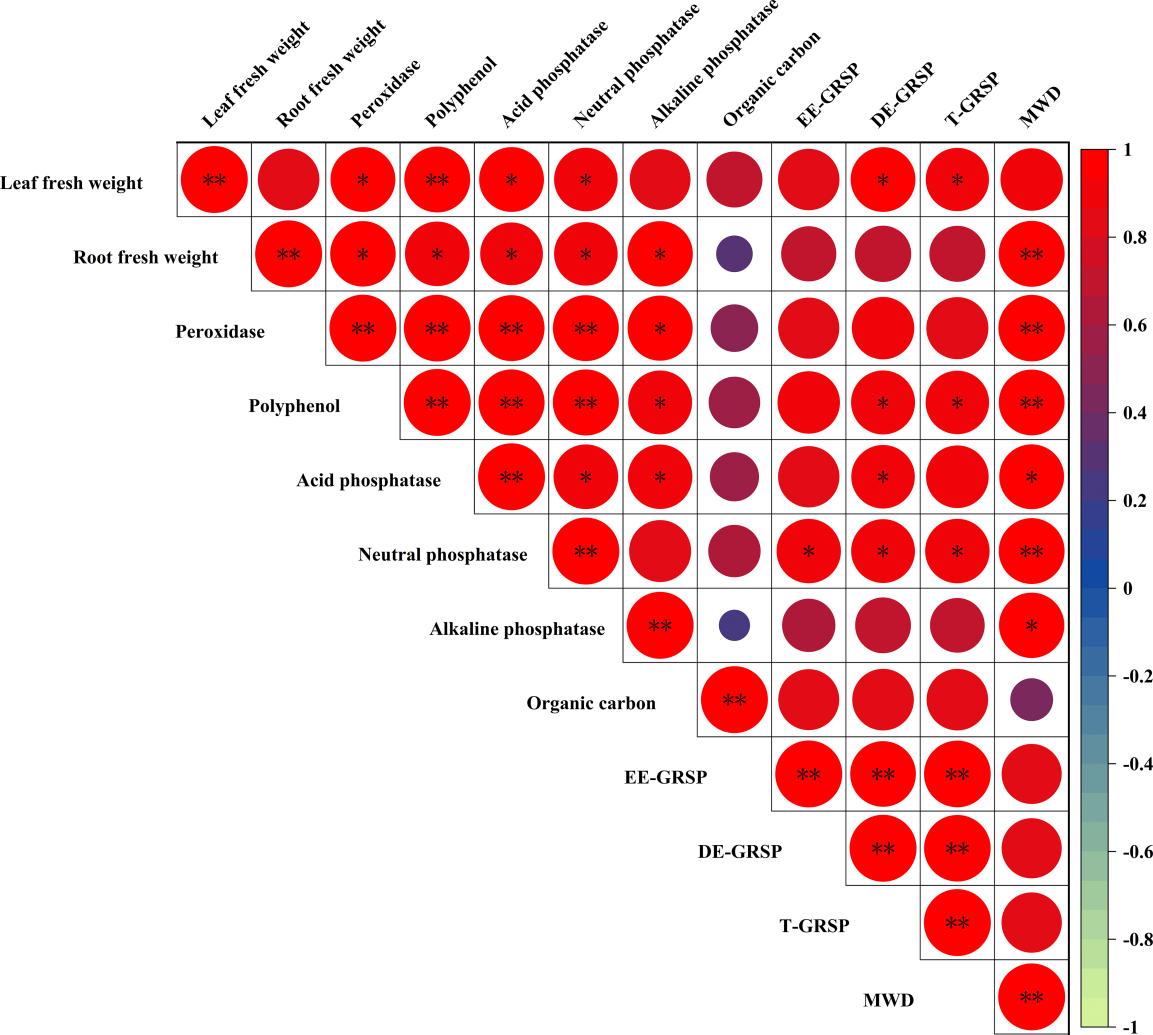
Pearson correlation matrix of biomass, rhizosphere soil enzyme activities (peroxidase, polyphenol, phosphatases), GRSP fractions (EE-GRSP, DE-GRSP), soil organic carbon (SOC), and mean weight diameter (MWD) in kumquat seedlings after 12 weeks. Red icons denote positive correlations (* *P*<0.05; ** *P*<0.01). GRSP, glomalin-related soil protein; EE-GRSP, easily extractable GRSP; DE-GRSP, difficultly extractable GRSP; SOC, soil organic carbon; MWD, mean weight diameter.

The MWD demonstrated significant positive correlations with root fresh weight, POD activity, polyphenol content, and neutral phosphatase activity (*P*<0.01). Additionally, MWD was positively correlated with both acid and alkaline phosphatase activities (*P*<0.05). Root fresh weight exhibited positive correlations with POD, polyphenol, and all three phosphatase activities (acid, neutral, and alkaline) (*P*<0.05). Leaf fresh weight showed positive correlations with DE-GRSP, T-GRSP, POD, acid and neutral phosphatase activities (*P*<0.05), and a significant positive correlation with polyphenol activity (*P*<0.01) (**Figure 6**).

## 4 Discussion

The study systematically investigated the dose-dependent effects of exogenous EE-GRSP on kumquat (*F. japonica*) seedling growth, root development, and rhizosphere soil properties. The findings identified an optimal concentration of 0.020 mg·mL^–1^ (3/4 strength) that effectively triggered synergistic soil-plant feedbacks, providing robust support for the hypothesis that exogenous EE-GRSP enhances plant performance by concomitantly improving both soil physical structure and biochemical processes. Specifically, this optimal EE-GRSP dosage significantly boosted kumquat biomass (leaf fresh weight: +18.29%, root fresh weight: +48.37%), optimized root system architecture (total root length +81.07%, root volume +66.81%, second-order lateral roots: +238.82%), enhanced SOC content (+50.09%), increased WSA proportion (2–4 mm fraction +109.12%), elevated MWD (from 0.42mm to 0.81mm), and stimulated key soil enzyme activities (PPO: +63.96%, POD: +39.26%, acid phosphatase: +46.15%). This comprehensive suite of improvements highlights EE-GRSP’s capacity to orchestrate a robust rhizosphere environment conducive to vigorous plant growth, initiating a positive cascade of inter-connected interactions.

The primary mechanism underpinning the observed growth enhancements was the profound optimization of soil physical structure by exogenous EE-GRSP. Consistent with its known properties as a powerful cementing agent akin to humic substances (Schindler et al., 2007), EE-GRSP significantly enhanced soil aggregation. At the optimal 3/4 concentration, MWD increased by 92.86%, and the critical 2–4 mm WSA was more than doubled relative to the control. This improved aggregate stability, corroborated by the strong positive correlations between MWD and both T-GRSP and EE-GRSP (r = 0.89, *P*<0.01; **Figure 6**), directly facilitating the formation of a stable, porous rhizosphere. Such an environment reduces mechanical impedance for root growth, enhances aeration, and optimizes water retention, thereby creating ideal conditions for root proliferation (Wang et al., 2015; Holátko et al., 2021; Guo et al., 2023). Consequently, root development was maximally stimulated at this dosage. However, a critical threshold was observed: at the full-strength EE-GRSP concentration (0.027 mg·mL^–1^), MWD declined to 0.62mm, and the 2–4 mm WSA fraction dropped, accompanied by a less pronounced increase in root fresh weight (+17.75%) compared to the 3/4 treatment. The precise mechanisms for this diminished efficacy, particularly given the concurrent peak in SOC, remain to be fully elucidated, but several plausible, non-mutually exclusive hypotheses can be proposed. One hypothesis involves detrimental physical changes: excessive EE-GRSP may have led to over-aggregation or clogging of soil pores, reducing aeration and root penetration. While this study lacks direct measurements of soil porosity (e.g., bulk density) to confirm this, it remains a plausible physical constraint. An alternative, yet complementary, hypothesis centers on adverse microbial responses. The sudden influx of a large amount of labile carbon (as evidenced by the peak in SOC) could have stimulated a rapid microbial bloom, leading to intense competition for other nutrients (i.e., nutrient immobilization) or a shift in the microbial community composition (Kuzyakov and Blagodatskaya, 2015). At sub-optimal concentrations (1/4 and 1/2 strength), MWD values (0.55mm and 0.64mm, respectively) indicated insufficient structural improvement to fully support significant root development, reinforcing the 3/4 concentration as the optimal point for initiating positive soil physical changes.

Building upon this enhanced physical foundation, exogenous EE-GRSP profoundly elevated soil biochemical activity, which is crucial for nutrient cycling and availability. Indeed, the improved soil structure itself likely contributed to this biochemical uplift by creating more favorable micr-ohabitats for microbial activity and enzyme stability through better aeration and moisture conditions (Kumar et al., 2017; Wilpiszeski et al., 2019). Rhizosphere enzyme activities, including PPO, POD, and acid phosphatase, exhibited a parabolic response, peaking at the 3/4 concentration with increases of 63.96%, 39.26%, and 46.15%, respectively. These enzymes are indispensable for biogeochemical cycling: PPO aids in lignin degradation and carbon solubilization (Ander and Eriksson, 1976), while acid phosphatase mineralizes organic phosphorus (Wang et al., 2018). This biochemical stimulation is likely driven by two further mechanisms. Firstly, EE-GRSP, as an exogenous organic macromolecule, directly contributed to the soil’s carbon pool (SOC+50.09% at 3/4 strength), providing an energy source for microbial metabolism and enzyme production (Holátko et al., 2021). Secondly, increased *de novo* EE-GRSP and T-GRSP levels contributed to stable GRSP-organic carbon complexes, acting as a slow-release carbon reservoir sustaining microbial activity (Wu et al., 2014). However, at the full EE-GRSP concentration, despite a remarkable 123.69% increase in SOC, enzyme activities declined. This suggests that while more carbon was introduced, it might have led to microbial metabolic imbalance or nutrient saturation, reducing nutrient release efficiency (Johnson and Loeppert, 2006; Wu et al., 2018). Lower EE-GRSP concentrations yielded only modest increases in enzyme activities and SOC, insufficient for optimizing nutrient cycling.

**Figure 7:**
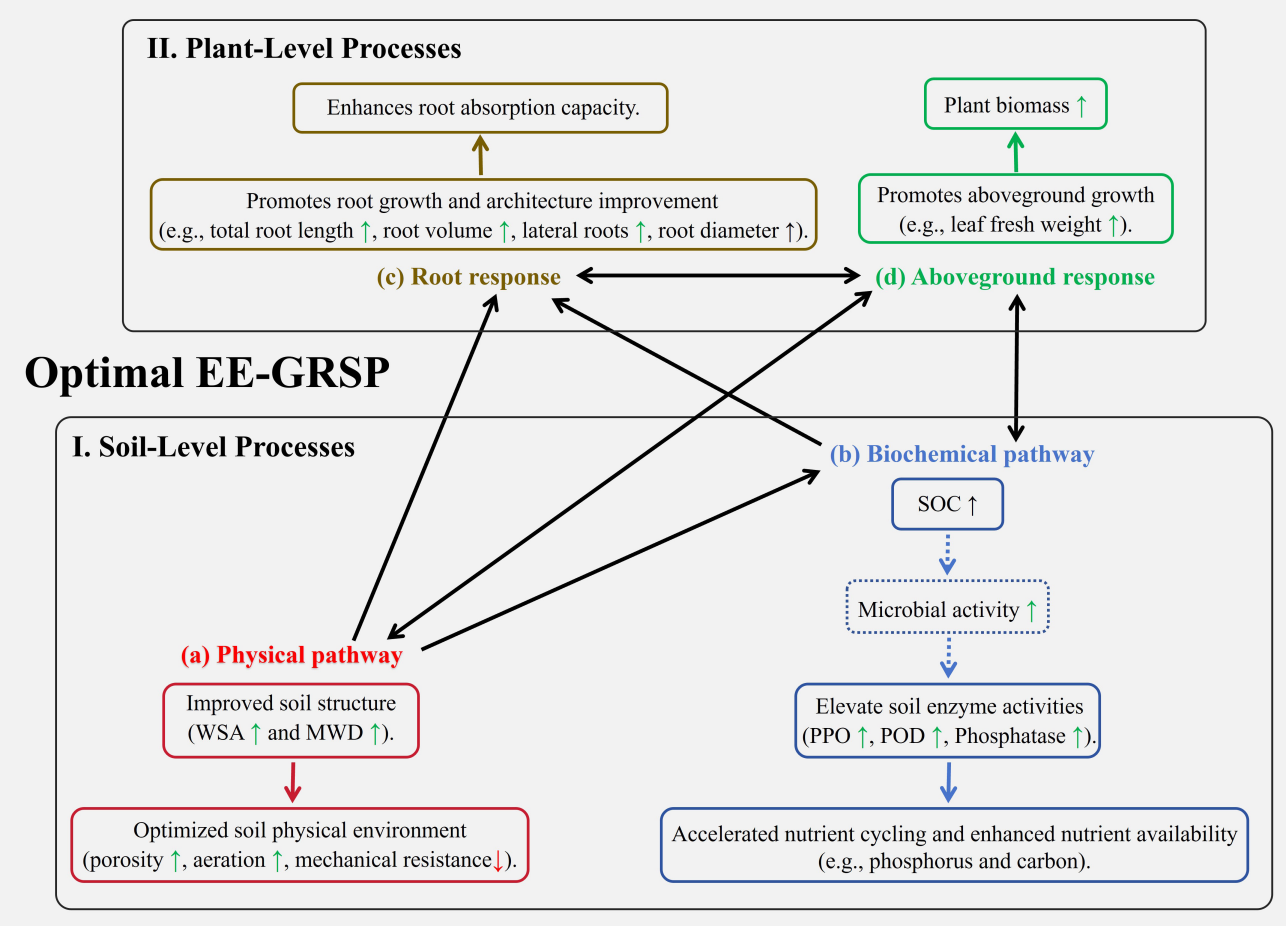
Conceptual model illustrating the synergistic soil-plant feedbacks promoted by optimal exogenous easily extractable glomalin-related soil protein (EE-GRSP) application in kumquat seedlings. The model depicts two interconnected levels: (I) soil-level processes and (II) plant-level processes. At the soil level, EE-GRSP initiates improvements through **(A)** a physical pathway, enhancing soil structure as indicated by increased water-stable aggregates (WSA) and mean weight diameter (MWD), which optimizes the soil physical environment (e.g., porosity, aeration, reduced mechanical impedance); and **(B)** a biochemical pathway, increasing soil organic carbon (SOC), potentially stimulating microbial activity, and boosting soil enzyme activities (polyphenol oxidase - PPO, Peroxidase - POD, and acid phosphatase), thereby accelerating nutrient cycling and availability. These soil improvements positively influence plant processes. At the plant level, **(C)** the enhanced soil physical and biochemical conditions promote root system development (increased total length, volume, lateral roots, and diameter) and absorption capacity. **(D)** This improved root function supports greater shoot growth (leaf fresh weight) and overall biomass accumulation. Solid boxes represent parameters or processes directly measured or confirmed in this study, while dashed boxes indicate inferred or potential processes. Single arrows denote a positive influence or causal progression (e.g., soil improvements leading to better root growth), while double arrows signify bidirectional feedback interactions (e.g., between enhanced biochemical activity and shoot growth, and between root and shoot development), highlighting the integrated nature of the soil-plant system response to optimal EE-GRSP treatment.

The synergistic improvements in soil physical and biochemical properties directly translated into enhanced physiological responses, particularly optimized root system architecture, which is pivotal for nutrient and water acquisition and forms a key component of the positive soil-plant feedback loop. At the 3/4 EE-GRSP concentration, root system modifications were maximized (total root length +81.07%, root volume +66.81%, average root diameter +18.84%). This optimized root development is a direct consequence of the favorable rhizosphere environment created by reduced mechanical stress (MWD+92.86%) and enhanced nutrient availability. Specifically, the vastly expanded root system inherently increases the plant’s capacity for nutrient foraging, while the concurrently stimulated phosphatase activity (+46.15% for acid phosphatase) directly enhances the bioavailability of soil phosphorus, a critical limiting nutrient. Crucially, a more extensive and metabolically active root system would, in turn, increase rhizodeposition, further enriching SOC (as observed with the 50.09% increase) and providing essential substrates that could contribute to ongoing aggregate stabilization and microbial activity, thereby reinforcing the beneficial soil conditions (**Figure 7**). This reciprocal enhancement underpins the substantial increases in root fresh weight (+48.37%) and overall leaf fresh weight (+18.29%). Conversely, at the full EE-GRSP concentration, attenuated root growth was likely attributable to reduced soil porosity or potential nutrient imbalances, indicating a disruption of this positive feedback. Suboptimal EE-GRSP concentrations resulted in limited root improvements, demonstrating insufficient soil optimization to elicit a strong plant growth response or fully engage the feedback mechanism.

It is noteworthy that the weekly application of EE-GRSP, a carbon- and nitrogen-rich protein solution, represents an input of organic N and C, which could contribute to the observed enhancements. However, several lines of evidence suggest that the unique functions of GRSP, rather than its nutrient content alone, were the primary drivers. Most compellingly, the distinct parabolic dose-response curve, where the full-strength treatment (providing the highest nutrient input) yielded diminished returns compared to the optimal 3/4 strength, is inconsistent with a simple fertilization effect. Furthermore, a quantitative estimation reveals the nutrient input was modest; at the optimal 3/4 strength, a total of approximately 3.2 mg of C and 1.0 mg of N were added to 1.5kg of soil over four weeks. This amount is unlikely to be the sole driver of the substantial biomass gains and the profound 92.86% increase in soil MWD. Instead, these effects, along with the strong correlation between GRSP concentration and aggregate stability, point directly to GRSP’s well-documented role as a potent soil aggregating agent. Therefore, while the added organic C and N likely provided supplementary benefits by serving as a microbial substrate, the unique structural and biochemical enhancements are more plausibly attributed to the specific physicochemical properties of the GRSP protein itself.

In summary, the concentration-dependent effects of exogenous EE-GRSP clearly establish a threshold for optimal efficacy, with the 3/4 concentration (0.020 mg·mL^–1^ protein) consistently maximizing plant biomass, root architecture, enzyme activities, and soil aggregate stability. This comprehensive suite of interconnected improvements provides compelling evidence that exogenous EE-GRSP enhances kumquat growth by synergistically optimizing both the physical and biochemical properties of the rhizosphere, thereby fostering a robust positive soil-plant feedback loop. The observed parabolic response (**Figure 4**) unequivocally demonstrates that benefits diminish beyond this optimum, likely due to excessive EE-GRSP leading to detrimental physical changes (e.g., over-cementation) or uncoupled biochemical processes (e.g., high SOC but low enzyme activity), thereby disrupting the delicate balance required for sustained benefits. This critical insight into the precise dose-response relationship of EE-GRSP provides valuable guidance for its practical application as a sustainable soil amendment.

While this study provides compelling evidence for the beneficial effects of optimal exogenous EE-GRSP application, certain limitations warrant consideration. First and foremost, a significant limitation is the absence of data on plant tissue nutrient concentrations and nutrient use efficiency. Such data are crucial to definitively establish the mechanistic link between the improved soil properties, root architecture, and the actual nutritional status of the plant. While our findings on enhanced phosphatase activity and root proliferation strongly imply an improved nutrient acquisition capacity, direct quantification of nutrient uptake is required to confirm this relationship. Second, the use of autoclaved soil and mycorrhiza-free seedlings under controlled potted conditions, while necessary to isolate the direct effects of exogenous EE-GRSP, may have underestimated the complex interplay with native microbial communities and AM fungal colonization in a natural environment. The intriguing anomaly at full concentration, where high SOC was observed but with reduced growth and enzyme activity, demands further investigation into the precise mechanisms of carbon utilization efficiency and microbial community shifts. Finally, our methodology could not distinguish between the residual GRSP applied exogenously and the GRSP produced *de novo*. Although the increase in the DE-GRSP fraction provides strong indirect evidence for new synthesis and transformation, this remains a key ambiguity.

Future research should therefore focus on: (1) validating the observed optimal EE-GRSP concentration and its long-term effects under diverse field conditions, while incorporating plant nutritional analysis to confirm nutrient uptake mechanisms; (2) specifically investigating the mechanisms behind the sub-optimal effects at supra-optimal concentrations by directly measuring soil physical properties (e.g., bulk density, porosity) and microbial community dynamics; (3) employing isotopic labeling techniques (e.g., using ^15^N-labeled GRSP) to definitively trace the fate of the applied protein and quantify true *de novo* production; and (4) developing and evaluating of efficient field delivery methods (e.g., fertigation or slow-release granular formulations) to refine practical application strategies.

## 5 Conclusion

This study definitively demonstrated that exogenous EE-GRSP, particularly at a three-quarters concentration (0.020 mg·mL^–1^), optimally enhances kumquat seedling growth and biomass accumulation through the establishment of robust synergistic soil-plant feedbacks (**Figure 7**). This optimal application effectively improved soil physical properties by significantly enhancing aggregate stability and MWD, while concomitantly stimulating key rhizosphere enzyme activities and increasing SOC and GRSP content. These comprehensive soil enhancements created a highly favorable rhizosphere environment, directly supporting accelerated root development and improved nutrient uptake, thereby maximizing plant growth responses. The findings highlight that the three-quarters dosage represents a critical balance for structural stability and nutrient availability, as excessive EE-GRSP application diminished benefits, likely due to detrimental soil physical changes or microbial constraints. Collectively, these results not only advance our mechanistic understanding of glomalin-mediated soil-plant interactions but also establish EE-GRSP as a promising and valuable soil amendment for sustainable citrus cultivation practices. Future research should prioritize validating its long-term efficacy and dose-response across diverse field conditions and citrus species to refine its practical application.

## Data Availability

The original contributions presented in the study are included in the article/supplementary material. Further inquiries can be directed to the corresponding author.
